# Association between irritable bowel syndrome and Parkinson’s disease by Cohort study and Mendelian randomization analysis

**DOI:** 10.1038/s41531-024-00691-5

**Published:** 2024-03-28

**Authors:** Zhi-yun Wang, Dong-rui Ma, Meng-jie Li, Yuan-yuan Liang, Zheng-wei Hu, Shuang-jie Li, Chun-yan Zuo, Chen-wei Hao, Yan-mei Feng, Meng-nan Guo, Xiao-yan Hao, Yuan-li Guo, Ke-ke Ma, Li-na Guo, Chan Zhang, Yu-ming Xu, Cheng-yuan Mao, Chang-he Shi

**Affiliations:** 1 Department of Neurology, The First Affiliated Hospital of Zhengzhou University, Zhengzhou University, Zhengzhou, Henan China; 2 Henan Key Laboratory of Cerebrovascular Diseases, The First Affiliated Hospital of Zhengzhou University, Zhengzhou University, Zhengzhou, Henan China; 3 Institute of Neuroscience, Zhengzhou University, Zhengzhou, Henan China; 4 NHC Key Laboratory of Prevention and treatment of Cerebrovascular Diseases, The First Affiliated Hospital of Zhengzhou University, Zhengzhou University, Zhengzhou, Henan China

**Keywords:** Diseases, Risk factors

## Abstract

This study aimed to investigate the association between irritable bowel syndrome (IBS) and Parkinson’s disease (PD) utilizing prospective cohort study and Mendelian randomization. The dataset contained a substantial cohort of 426,911 participants from the UK Biobank, discussing the association between IBS and PD with Cox proportional hazards models and case-control analysis while adjusting for covariates such as age, gender, ethnicity and education level. In univariate Cox regression model, the risk of PD was reduced in IBS patients (HR: 0.774, 95%CI: 0.625–0.956, *P* = 0.017), but the statistical significance diminished in the three models after adjusting for other variables. In a few subgroup analyses, IBS patients are less likely to develop into PD, and patients diagnosed with IBS after 2000 also had a lower risk (HR: 0.633, 95%CI: 0.403–0.994, *P* = 0.047) of subsequently developing PD. In addition, we matched five healthy control participants based on gender and age at the end of the study for each IBS patient diagnosed during the follow-up period, and logistic regression results (OR:1.239, 95%CI: 0.896–1.680, *P* = 0.181) showed that IBS was not associated with the risk of PD. Mendelian randomization did not find significant evidence of the causal relationship between IBS and Parkinson’s disease (OR: 0.801, 95%CI: 0.570–1.278, *P* = 0.204). Overall, we suggest that IBS status is not associated with the risk of developing PD, and that these findings provide valuable insights into the clinical management and resource allocation of patients with IBS.

## Introduction

Parkinson’s disease (PD) ranks as the second most prevalent neurodegenerative disorder globally, trailing only Alzheimer’s disease, with an estimated 1-2% prevalence among individuals aged 65 years and older^1,2^. Its primary clinical manifestations encompass resting tremor, bradykinesia and rigidity^3^. Notably, PD often exhibits a subtle onset, with symptoms emerging years before formal diagnosis. Moreover, non-motor symptoms such as cognitive dysfunction and gastrointestinal disturbances frequently precede motor symptoms, increasingly recognized as early harbingers of PD^4^. Neuropathologically, PD is characterized the loss of dopaminergic neurons in the substantia nigra and the presence of Lewy bodies. The dopamine depletion contributes to basal ganglia dysfunction, culminating in motor impairment^5,6^. While the precise etiology of PD remains elusive, current understanding implicates a multifaceted interplay between genetic and environmental factors^7,8^. Emerging evidence advises an association between PD and various gastrointestinal symptoms, possibly linked to alterations in gut microbiota, increased intestinal mucosal permeability and gut-brain axis communication^9,10^.

Irritable bowel syndrome (IBS) represents a common functional gastrointestinal disorder characterized by chronic or recurrent abdominal pain concomitant with changes in bowel habits, which can be ameliorated or exacerbated after defecation^11^. The pathogenesis of IBS likely involves a myriad of factors, including genetic predisposition, infectious triggers, dysbiosis of the intestinal microbiota, inflammatory responses and disruptions in intestinal mucosal integrity^12^. To diagnose IBS, other organic diseases that may present with similar symptoms must be meticulously excluded. A mounting body of research points to the potential involvement of gut microorganisms in the pathogenesis of IBS^12–15^. Additionally, prevalent neurobehavioral symptoms such as anxiety and depression suggest that IBS may be considered a gut-brain axis disorder^16^.

**Fig. 1:**
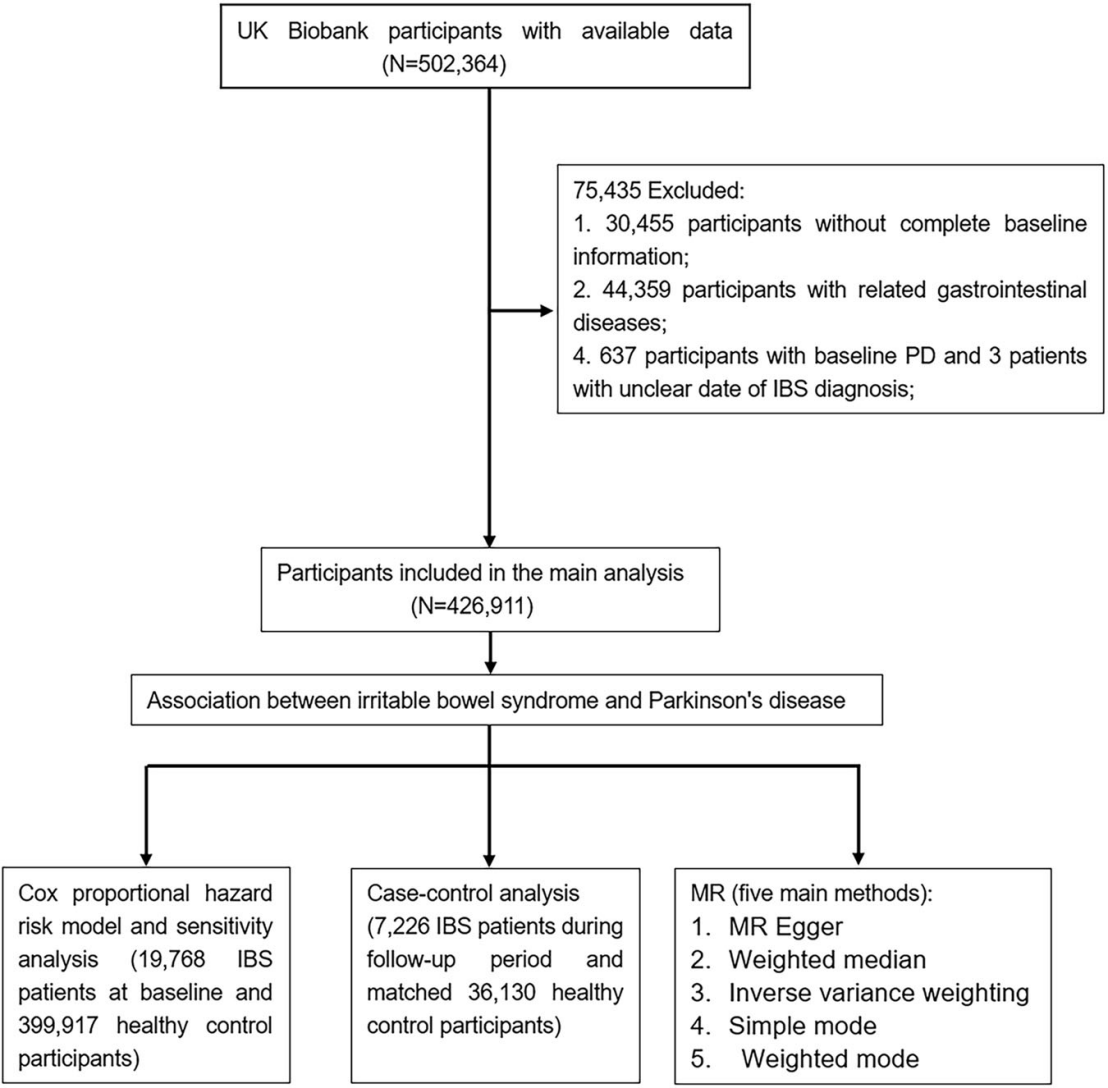
Analytical pipeline of the study. PD Parkinson’s disease, IBS Irritable bowel disease, MR Mendelian randomization.

Given the shared involvement of gut microbes and the gut-brain axis in both PD and IBS, a plausible correlation between the two conditions exists^17^. However, current studies examining this association are limited and exhibit conflicting results. To address this gap, we undertook a comprehensive large-scale cohort study using Cox proportional hazard model and logistic regression. Moreover, we employed Mendelian randomization (MR) analysis to validate the causal relationship between these two conditions, aiming to provide a more precise assessment of the interplay between IBS and PD (Fig. 1).

## Results

### Baseline characteristics

Among the total of 419,685 participants included in Cox proportional risk regression, 399,917(95.29%) participants were included in the control group, while 19,768(4.71%) patients were diagnosed with IBS before the baseline (Table 1). Compared to the control group, the IBS group had a higher proportion of females, a slightly lower frequency of alcohol intake and educational level, a higher proportion of non-smokers, poorer overall health and a higher prevalence of long-term illness, disability, or frailty. The mean (SD) follow-up duration was 13.93 years (±2.09) and 14.08 years (±1.83) for the IBS and non-IBS groups respectively. In the case-control analysis, 7,226 IBS patients and 36,130 healthy controls were nearly identical in age and gender (Supplementary Table 4).

**Table 1:** Baseline characteristics and incidence of Parkinson’s disease

Variables	Levels	No IBS (*N* = 399,917)	IBS (*N* = 19,768)	*P* Value
Age	Mean ± SD	56.33 ± 8.10	56.03 ± 7.92	**<0.001**
Gender	Female	211,680 (52.9%)	14,628 (74%)	**<0.001**
	Male	188,237 (47.1%)	5140 (26%)	
Ethnicity	Non-white	20,826 (5.2%)	596 (3%)	**<0.001**
	White	379,091 (94.8%)	19,172 (97%)	
Education	College	134,464 (33.6%)	6205 (31.4%)	**<0.001**
	Non-College	265,453 (66.4%)	13,563 (68.6%)	
Alcohol Intake	0	73,080 (18.3%)	4502 (22.8%)	**<0.001**
	1	44,057 (11%)	2573 (13%)	
	2	104,226 (26.1%)	5059 (25.6%)	
	3	95,306 (23.8%)	4184 (21.2%)	
	4	83,248 (20.8%)	3450 (17.5%)	
Smoking Status	Never	221,455 (55.4%)	11,205 (56.7%)	**<0.001**
	Previous	137,259 (34.3%)	6692 (33.9%)	
	Current	41,203 (10.3%)	1871 (9.5%)	
BMI	18.5-24.9	127,123 (31.8%)	6908 (34.9%)	**<0.001**
	<18.5	1905 (0.5%)	154 (0.8%)	
	25-29.9	173,217 (43.3%)	7963 (40.3%)	
	>30	97,672 (24.4%)	4743 (24%)	
TDI	Mean ± SD	−1.38 ± 3.05	−1.46 ± 2.99	**<0.001**
Long standing illness	0	285,740 (71.4%)	11,529 (58.3%)	**<0.001**
	1	114,177 (28.6%)	8239 (41.7%)	
Overall health rating	Poor	13,908 (3.5%)	1435 (7.3%)	**<0.001**
	Fair	77,423 (19.4%)	5554 (28.1%)	
	Good	236,985 (59.3%)	10,936 (55.3%)	
	Excellent	71,601 (17.9%)	1843 (9.3%)	
PRS	Low Risk	100,304 (25.1%)	5058 (25.6%)	0.278
	Medium Risk	200,058 (50%)	9820 (49.7%)	
	High Risk	99,555 (24.9%)	4890 (24.7%)	
PD	0	397,596 (99.4%)	19,678 (99.5%)	0.026
	1	2321 (0.6%)	90 (0.5%)	
time	Mean ± SD	13.93 ± 2.09	14.08 ± 1.83	<0.001

Continuous variables are shown as means and standard deviations, categorical variables are shown as percentages, while baseline characteristics of IBS and non-IBS groups are compared using ANOVA for continuous variables and Chi-square tests for categorical variables, with *P*-values < 0.001 highlighted in bold font. TDI, Townsend Deprivation Index.

### Cox proportional hazard model analysis

**Fig. 2:**
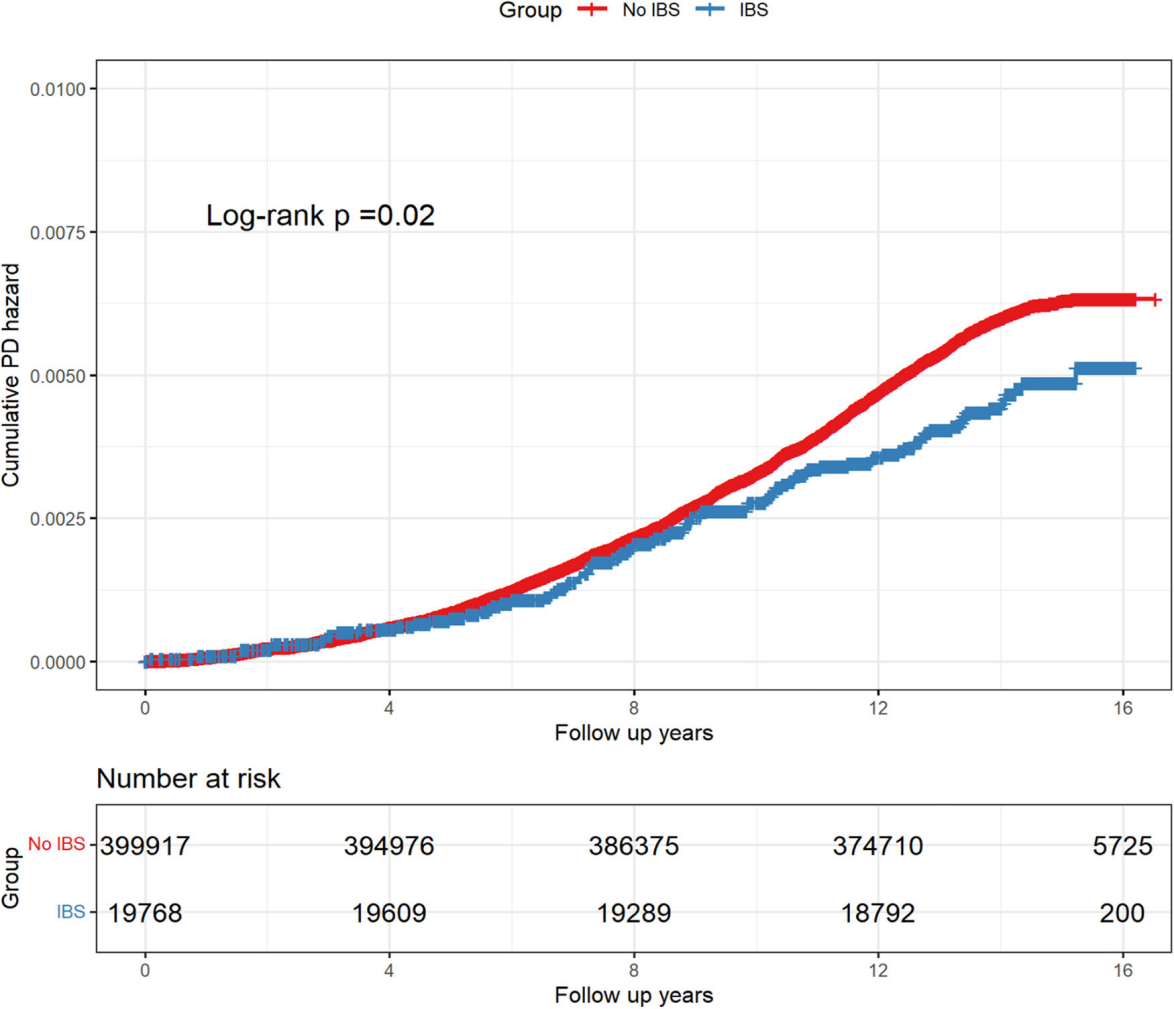
Kaplan-Meier plot for the cumulative probability of PD risk.

Using the Kaplan-Meier method (Fig. 2), we plotted the cumulative probability of PD between the two groups and tested for differences using the log-rank test (*P* = 0.02). The assumption of equal proportional hazards was satisfied (*P* = 0.44), indicating that the IBS group had a lower risk of PD compared to the non-IBS group (HR: 0.774, 95%CI: 0.627–0.956, *P* = 0.017). During the follow-up period, 2,321 participants in the non-IBS group and 90 in the IBS group were diagnosed with PD. The incidence rate was 4.17 and 3.23 per 10,000 person-years in healthy control group and IBS group respectively, resulting in a relative incidence rate of 1.28 (95% CI: 1.04–1.59). In the univariate Cox regression analysis, IBS status and 11 covariates were statistically significant (Supplementary Figure 1), particularly older age (HR: 1.147, 95% CI: 1.139–1.156), male population (HR: 2.092, 95% CI: 1.926–2.273) and the presence of long-standing illness, disability or frailty (HR: 1.650, 95% CI: 1.520–1.790). Additionally, individuals who drank alcohol with high frequency showed a tendency of reduced PD risk compared to those who never drank or drank only on special occasions. However, for people who drank every day or almost every day, this tendency disappeared.

**Fig. 3:**
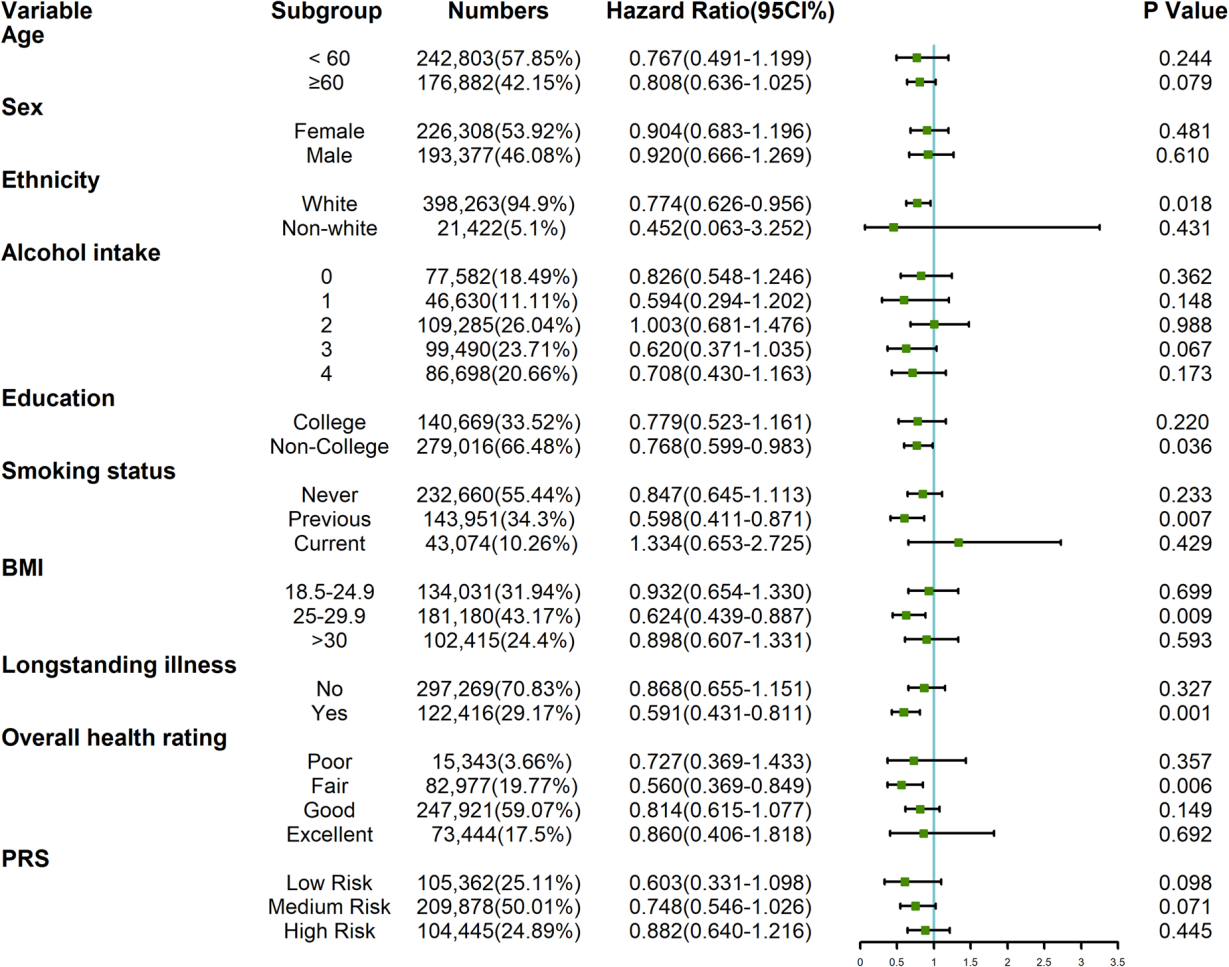
Forest plot for subgroup analysis.

**Fig. 4:**
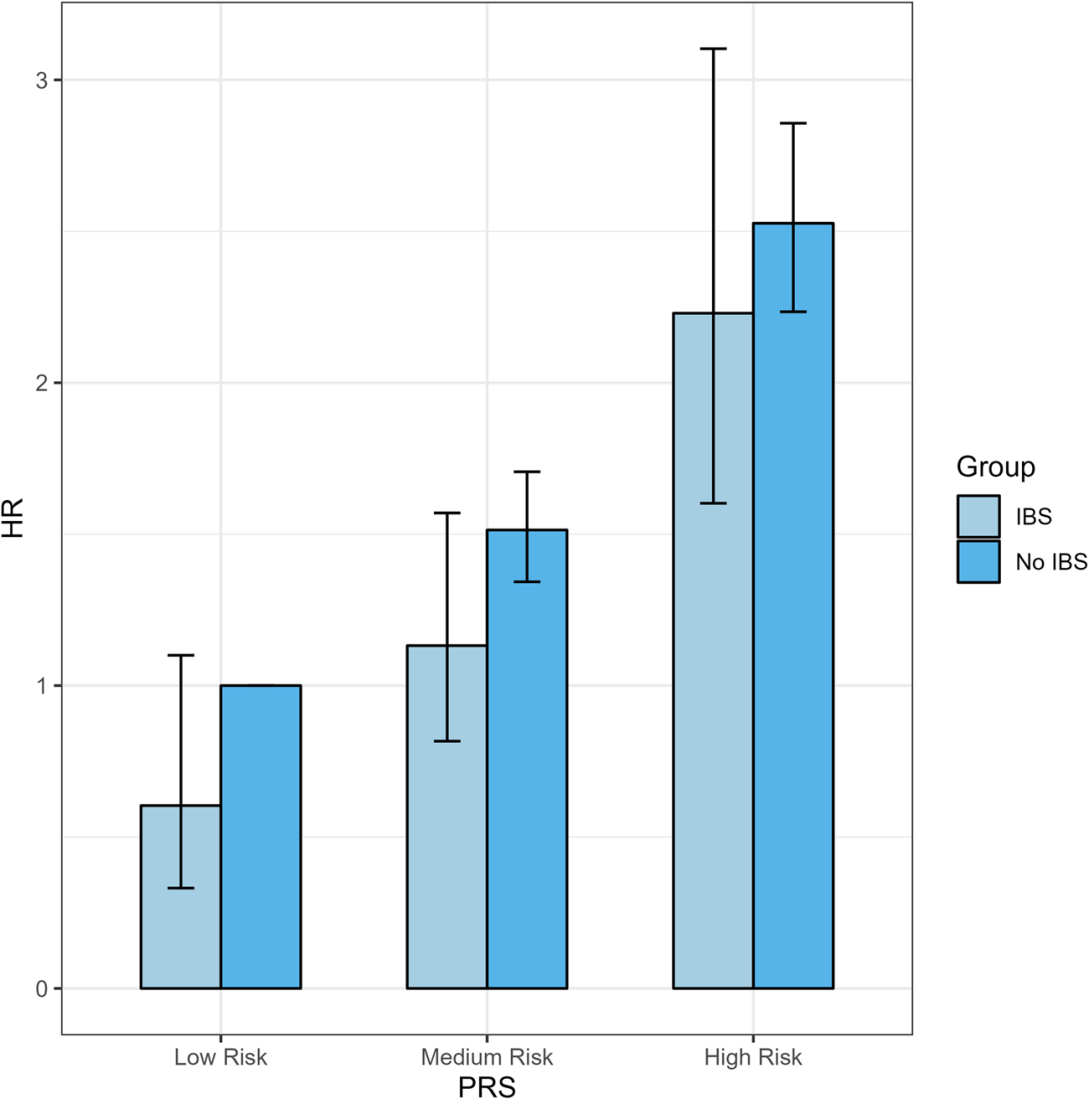
Risk of PD according to PRS and IBS status. PRS polygenic risk score, HR Hazard Ratio.

**Fig. 5:**
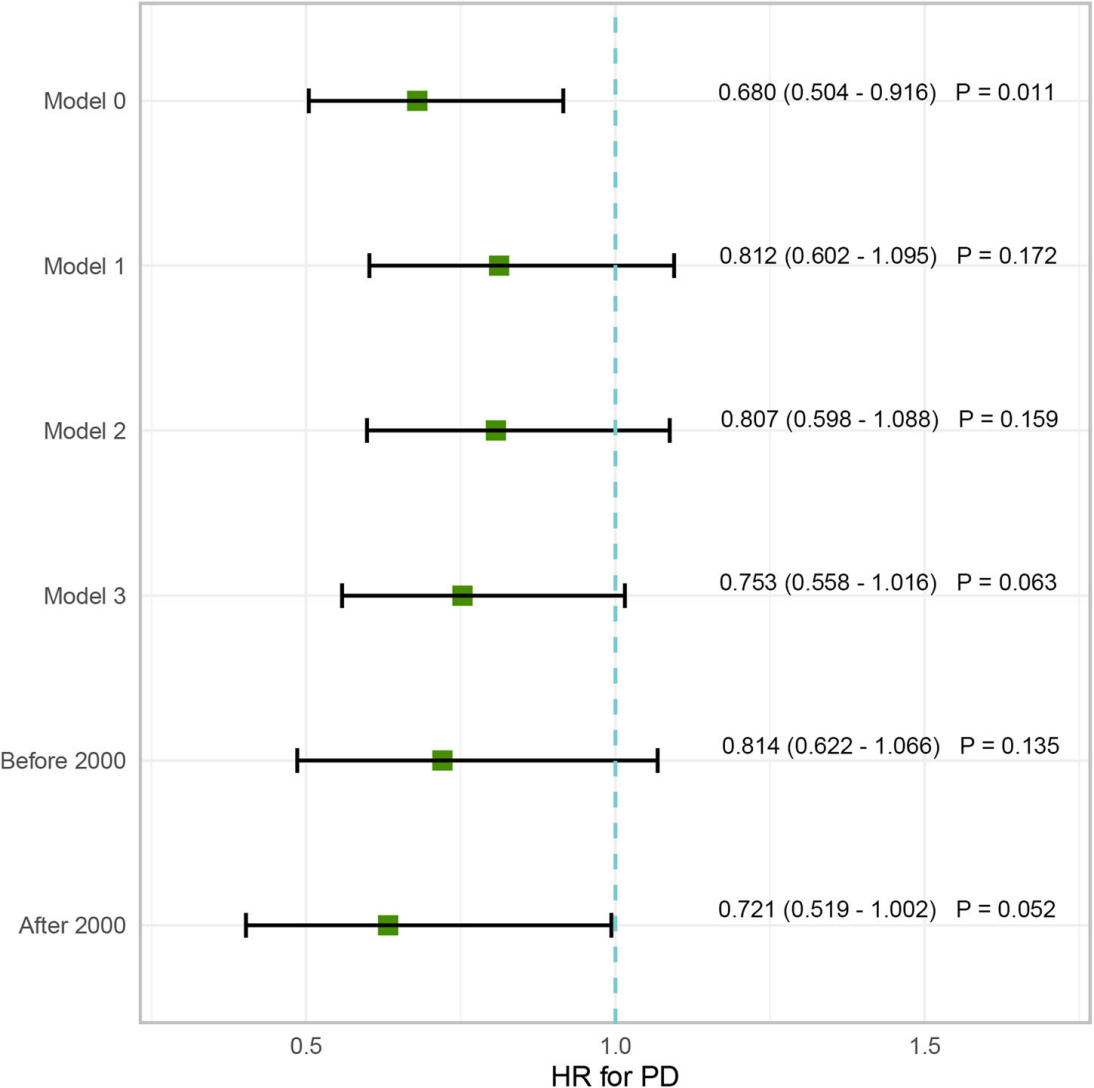
Sensitivity analysis between IBS and PD. Model 0, univariate Cox regression; Model 1 adjusted age, gender, and ethnicity; education level, drinking, smoking status, BMI, and Townsend deprivation index were further included in Model 2; all variables were included in Model 3. ‘Before 2000’ and ‘After 2000’ respectively represent the time of IBS diagnosis.

Univariate Cox regression analysis indicated that IBS was associated with a lower risk of PD. However, after adjusting for other covariates in different models (Table 2), IBS did not seem to be associated with PD in any of the three models. Subgroup analysis (Fig. 3) stratified by age and categorical variables revealed that IBS continued to be associated with reduced PD risk in the White ethnicity group (HR: 0.774, 95% CI: 0.626–0.956, *P* = 0.018), participants with a previous history of smoking (HR: 0.598, 95% CI: 0.411–0.871, *P* = 0.007), BMI of 25–29.9 (HR: 0.624, 95% CI: 0.439–0.887, *P* = 0.009) and those with non-college education (HR: 0.768, 95% CI:0.599–0.983, *P* = 0.036). Individuals with long-term illness, disability, or frailty (HR: 0.591, 95% CI:0.431–0.811, *P* = 0.001) or fair overall health rating (HR: 0.560, 95% CI: 0.369–0.849, *P* = 0.006) also demonstrated that IBS was linked to a decreased risk of PD. Subsequently, we created an interaction term (Fig. 4) combining the prevalence status of IBS and the polygenic risk score (PRS) of PD, and we observed a significantly increased risk of PD in the non-IBS group with high PRS (HR: 2.230, 95% CI: 1.602–3.103, *P* < 0.001). In sensitivity analysis (Fig. 5), after excluding self-reported IBS patients, the results from Cox proportional hazard regression remained in alignment with our primary findings, indicating that IBS may reduce the risk of PD (HR: 0.680, 95% CI:0.504–0.916, *P* = 0.011). However, once we adjusted for other variables, the statistical significance between the two diseases disappeared. Furthermore, among IBS patients diagnosed after the year 2000, our observations indicated a decline in the risk of PD associated with IBS (HR: 0.633, 95% CI:0.403–0.994, *P* = 0.047).

**Table 2:** Multivariate analysis for incidence of PD in participants with IBS

	HR	95%CL	*P* value
Model 1	0.951	0.770–1.175	0.644
Model 2	0.943	0.763–1.165	0.588
Model 3	0.872	0.706–1.079	0.207

Model 1 adjusted age, gender, and ethnicity; education level, drinking, smoking status, BMI, and Townsend deprivation index were further included in Model 2; all variables were included in Model 3.

### Case-control study

Of the 43,356 participants, there were a total of 247 patients diagnosed with PD. The occurrence of IBS did not reduce the risk of PD (OR:1.239, 95%CI: 0.896–1.680, *P* = 0.181) when age and gender between the IBS group and the matched group were almost identical. Although not statistically significant, the probability of PD occurrence was higher in the IBS group than in the healthy control group, which is in contrast to the results of the Cox proportional hazard model analysis.

### Mendelian randomization

The results from the five principal Mendelian randomization (MR) methods (Table 3) (Supplementary Figure 2-5) provided additional validation to the conclusions derived from our previous Cox regression analysis. After MR-PRESSO testing the horizontal pleiotropy, we removed the outlier rs13321176, the MR analysis incorporated a total of 16 single nucleotide polymorphisms (SNPs). The results obtained by the main IVW method (OR: 0.801, 95% CI: 0.570–1.278, *P* = 0.204) suggested the absence of a causal relationship between IBS and PD. In addition, there was no horizontal pleiotropy among the 16 instrumental variables (IVs) (P(MR-PRESSO) = 0.12) and no heterogeneity (*P* (MR Egger) = 0.114, *P*(IVW) = 0.077). In addition, in the context of harmonizing the versions of the reference genome sequences, we compared the risk loci associated with PD identified by Nalls et al with the Single Nucleotide SNPs utilized in current analysis^18^, and found no overlap.

**Table 3:** MR estimates from each method of assessing the causal effects of IBS on PD

Method	N(SNPs)	Odds ratio(95% CI)	*P* value
MR Egger	16	0.072(0.002–2.254)	0.157
Weighted median	16	0.821(0.546–1.125)	0.344
Inverse variance weighted	16	0.801(0.569–1.129)	0.204
Simple mode	16	0.827(0.378–1.811)	0.642
Weighted mode	16	0.834(0.391– 1.779)	0.646

## Discussion

It is essential to note that PD is typically diagnosed when motor symptoms appear, by which time approximately 60% of the patient’s substantia nigra neurons have already been lost^19^. Therefore, understanding the role of prodromal symptoms in the pathological progression of PD, such as gastrointestinal dysfunction, holds significant importance for early detection and the development of neuroprotective strategies^5^. In terms of PD, gastrointestinal non-motor symptoms are almost universally present, including salivation, dysphagia, constipation and changes in stool characteristics^20^. As one of the most common intestinal disorders, the exact mechanism by which IBS interacts with PD remains unclear. However, factors associated with IBS, such as low-grade mucosal inflammation, immune activation disorders, intestinal permeability changes, metabolic abnormalities and neuroendocrine system disorders, might involve some fundamental elements of PD^21^. These factors might make the gastrointestinal tract more vulnerable to pathogens, potentially leading to it becoming the initial site for α-synuclein (αSYN) accumulation. Moreover, the gut-brain axis plays a central role in both IBS occurrence and the hypothesis that PD may originate from the intestine^22^. Disturbance in intestinal function and dysregulation of gut microbiota may trigger local and systemic inflammatory responses and gut glial cell activation, ultimately leading to the development of αSYN pathology^23^. A study evaluated the relationship between gastrointestinal αSYN pathology and gastrointestinal dysfunction in PD patients and neurointegrity subjects, and the results showed that regardless of PD or not, αSYN pathology of gastric and colon mucosa was not correlated with gastrointestinal symptoms, indicating that deposition of αSYN in the muco-enteric nervous system may not be reflected in functional impairment of the affected portion of the intestine^24^. However, further clinical evidence is required to confirm this association and examine possible reverse causation.

Similar with a previously reported study, a Finland-based retrospective follow-up study that included 28,150 IBS patients and 98,789 non-IBS controls during 1998-2014 showed that the risk ratio between IBS and non-IBS groups was not constant over longer follow-up periods, only in the first two years of follow-up, patients with IBS had a higher risk of developing PD^25^. However, previous systematic reviews and meta-analyses have shown an association between IBS and an increased risk of PD^26–28^. In the Taiwanese population, patients with IBS have a 48% higher risk of PD than non-IBS patients^29^, and a nested case-control study from Swedish and a nationwide population‑based matched‑cohort study from Korea came to the similar conclusion^30,31^.

However, most of these studies were case-control or cross-sectional studies, with few baseline IBS cases coming from cohort studies to further clarify the relationship between IBS and PD. The study used data from the UK Biobank population cohort, which excluded participants with other gastrointestinal and neurological conditions at baseline or during follow-up, and took into account the interval between IBS or PD diagnosis and study inclusion, with a total of 426,911 participants included. In univariate Cox regression analysis, having IBS (HR: 0.774, 95%CI: 0.627–0.956, *P* = 0.017) was associated with reduced risk of PD compared to those without IBS, but this association was not significant in any of the three multivariate models adjusted for different covariates. Subgroup analysis indicated that IBS showed a tendency to reduce the risk of PD in certain populations, including White individuals, previous smokers, non-college-educated individuals, those with BMI in the range of 25–29.9, individuals with longstanding illness and those whose overall health rating is fair. Although the underlying reasons for this phenomenon are not fully understood, it is possible that IBS patients may adopt a healthier lifestyle due to their chronic and recurrent symptoms and increased medical attention. Nevertheless, the interaction between IBS and PD genetic risk was not significant, the cross-control analysis also found no association between IBS and PD. In the case of IBS, psychological stress is considered a significant contributing factor^32^, with up to one-third of patients experiencing comorbid anxiety and depression^33^, adding complexity to the clinical picture. In addition, in the early stages of PD, a reduction in dopamine levels can lead to the early onset of these symptoms^34^. Even in MR Analysis, we did not find the causal relationship between IBS and PD, which further verified the conclusion of Cox regression analysis. Through the analysis of 7,784,415 SNPs across 37,688 cases, 18,618 UK Biobank proxy cases, and 1,417,791 controls, Nalls et al identified a total of 90 independent genome-wide significant association signals^18^. By conducting a one-to-one comparison, we did not discover any loci that overlapped with the SNPs utilized in our analysis. While IBS patients may exhibit a heightened awareness of their health, the prolonged state of living with IBS, coupled with the intermittent bouts of constipation or diarrhea characteristic of IBS, could impart an elevated risk of developing PD^35,36^. Although our results do not support a causal relationship between IBS and PD, the specific mechanism warrants further investigation.

A significant advantage of our study is the use of a well-designed, large-scale prospective cohort with controls and a long follow-up period, enabling sufficient cases for investigating associated risks. Moreover, potential confounding factors were controlled by excluding other known digestive diseases at baseline. Additionally, the use of a national register for Parkinson’s detection ensures accuracy and reliability. The robustness of the results was further confirmed through MR analysis. However, there are some limitations in this study. Covariates that change over time may introduce bias, such as BMI, smoking status. In addition, although many potential confounders were carefully controlled, some unmeasured or unknown potential covariates could still confuse the association between IBS and PD. Although the risk of PD elevated with the increase of PRS in this population, PRS was only validated in 436 patients with PD. Finally, the identification of IBS relies on the ICD-10 code, which can lead to misclassification due to changes in diagnostic criteria during follow-up.

In conclusion, IBS is not associated with an overall increased risk of PD compared to non-IBS individuals, and that certain subgroups of the population with IBS may have a lower risk of developing PD. More studies are needed in the future to further understand the association between IBS and PD.

## Methods

### Study population

The study utilized data from the UK Biobank (UKB), a prospective cohort study that enrolled over 500,000 participants aged 40 to 69 years from 22 assessment centers in the UKB between 2006 and 2010, with a response rate of 5.47 percent. Participants provided comprehensive information, including personal details, lifestyle factors, health status, genotype data at baseline and during follow-up. Ethical approval for the UK Biobank study was obtained from the Northwest Multicenter Research Ethics Committee, and all participants provided written informed consent. The present analyses were conducted under UK Biobank application number 104811.

### Participant selection

Among the 502,364 participants in the UK Biobank (UKB), we initially excluded individuals with incomplete baseline data (*n* = 30,455), as well as those with other gastrointestinal and neurological disorders at baseline or during follow-up (*n* = 44,359) (Supplementary Table 1)^25^. After calculating the time interval between the diagnosis of IBS or PD and their inclusion in the study, we further excluded participants with PD before baseline (*n* = 637) and indeterminate dates (*n* = 2) for IBS diagnose. Consequently, all PD cases occurred after the diagnosis of IBS, a total of 426,911 participants were included in the final analysis.

### Definition of IBS and PD

The diagnosis of IBS (K58) and PD (G20) was ascertained based on the International Classification of Disease-10 (ICD-10) codes derived from various sources, including Death register, Primary care, Self-report and Hospital admissions data. In the overall population, there were a total of 26,994 IBS patients and 2,460 PD patients. Furthermore, we collated a comprehensive list of all utilized variables from the UKB (Supplementary Table 2).

### Covariates

Baseline characteristics of the participants were collected, including age (continuous variable), gender (male/female) and Townsend deprivation index (continuous variable). The Townsend deprivation index was calculated at the time of participants’ enrollment in the UK Biobank based on previous national census output areas, with each participant assigned a score corresponding to the output area where their postcode was located. Education level (college/non-college), ethnicity (white/non-white), smoking status, alcohol intake, overall health rating (poor/fair/good/excellent) and long-standing illness, disability or infirmity (0: no/1: yes) were also assessed via the Assessment center’s Touchscreen. Following UK Biobank classifications, we categorized individuals with British, Irish and other white backgrounds as ‘White’, while the remaining groups were considered ‘non-White’. Participants who responded as “Do not know” or “Prefer not to answer” were excluded from the analysis. Smoking status was categorized as “Never”, “Previous” and “Current”, while drinking status was represented as “0” for those who never drank or drank only on special occasions, “1” for drinking 1–3 times a month, “2” for drinking 1–2 times a week, “3” for drinking 3–4 times a week and “4” for drinking every day or almost every day. Body mass index (BMI) values were derived from participants’ height and weight measurements during their initial assessment center visit and categorized according to WHO standards as “18.5-24.9” (normal range), “<18.5” (underweight), “25-29.9” (overweight) and “>30” (obesity).

Compared with previous studies, we introduced the standard PRS from the UKB as one of the covariates. The PRS score combined with the whole genome information gives an individual genetic susceptibility to disease^37^. The UK Biobank has published a polygenic risk score for 53 diseases and quantitative traits, with a strong ability to stratified individual risk^38^. In our study, participants were assigned PRS based on their genomic data, with risk levels classified into three levels based on the top 25%, middle 50% and bottom 25% of all 502,364 participants (low risk, moderate risk and high risk). In total, 11 covariates were considered in this study.

### Statistical analysis

Baseline characteristics of the study population were presented as mean and standard deviation for continuous variables and as percentages for categorical variables. To compare the baseline characteristics between the IBS and non-IBS groups, analysis of variance was used for continuous variables, and chi-square tests were used for categorical variables.

Cox proportional hazards models were employed to assess the association between IBS and PD. The endpoint events considered in the models were the first diagnosis of PD, the end of the study (June 2023), loss to follow-up or death during the follow-up period. The time interval from baseline to endpoint events was calculated. Survival curves for the IBS and non-IBS groups were plotted using the Kaplan-Meier method, and differences were tested using the log-rank test. Univariate Cox regression models were constructed initially, and variables with statistical significance were incorporated into the multivariate Cox regression model, which was then used to map forest plot. Subsequently, we established three multivariable Cox regression models to assess the relationship between IBS and PD. Model 1 included age, gender and ethnicity as covariates. In Model 2, we further incorporated education level, alcohol intake, smoking status, BMI and the Townsend deprivation index. Finally, all variables were included in Model 3. Additionally, subgroup analysis and interaction tests were conducted. Interaction terms based on IBS status and PRS score were created to explore potential changes in the risk of PD when IBS status and genetic risk of PD were combined. Subgroup analysis was performed to investigate whether there were variations in the relationship between IBS and PD risk among different subgroups of participants.

In the sensitivity analysis, we systematically excluded a subset of participants (*n* = 8713) whose IBS diagnosis records relied on self-reported information and then conducted a repeated Cox proportional hazard regression. Moreover, given that a significant majority of participants had received their IBS diagnoses before the year 2000, we employed stratification for IBS participants based on their diagnosis dates, distinguishing them into ‘Before 2000’ (*n* = 5914) and ‘After 2000’ (*n* = 5141), to examine whether the duration of illness had any discernible impact on the incidence of PD.

In this longitudinal study, we then focused on individuals newly diagnosed with IBS after their initial baseline assessment. For 7226 IBS participants after baseline, we used propensity matching score to match each of them to 5 healthy controls based on gender and age at the end of follow-up to assess the potential relationship between IBS and PD.

### Mendelian randomization

To further explore the causal relationship between IBS and PD, we used MR, a genetic method for epidemiological studies^39^. Genotype data were utilized as instrumental variables, allowing for the assessment of causal relationships while minimizing the influence of confounding factors^40–42^. Five default methods, including MR Egger, weighted median, inverse variance weighting (IVW), simple mode and weighted mode, were employed in the MR analysis^43,44^. We primarily applied the IVW to obtain the MR estimate for each Wald estimate, and sensitivity analysis was conducted to ensure the validity of the results. The MR-PRESSO package was utilized for heterogeneity and pleiotropy tests to detect the presence of horizontal pleiotropy in exposure factors and outcomes, while the leave-one-out method was employed to assess the effect of individual SNPs by sequentially removing each SNP and examining the changes in MR results. For SNP selection (Supplementary Fig. 6), we initially selected them less than the genome-wide statistical significance threshold (1 × 10^−6^) as instrumental variables (IVs). In order to ensure no linkage disequilibrium (LD) imbalance among the included IVs, the clumping process (*R*^2^ < 0.01, clumping distance = 1000 kb) was employed in this study to assess the LD between the included SNPs. To avoid any potential distortion of strand orientation or allele coding, we excluded palindromic SNPs (rs1036958, rs2736155, rs541003), resulting in a final set of 17 IVs (Supplementary Table 3).

### Reporting summary

Further information on research design is available in the Nature Research Reporting Summary linked to this article.

### Supplementary information

Supplementary Data

Reporting Summary

## Data Availability

Data for Mendelian randomization are available from the open GWAS database and the GWAS catalog database, while other data supporting the results of this study are obtainable upon request to the UK Biobank.

## Code availability

The underlying code for this study is not publicly available but may be made available to qualified researchers on reasonable request from the corresponding author.
