# Supplementary material for: Association between irritable bowel syndrome and Parkinson’s disease by Cohort study and Mendelian randomization analysis
Source: NPJ Parkinsons Dis. 2024 Mar 28;10:70. doi: 10.1038/s41531-024-00691-5 (PMC10978991; doi:10.1038/s41531-024-00691-5)
Supplement: Supplementary file 1 — Supplementary Data [file 41531_2024_691_MOESM1_ESM.pdf]

List of Supplementary Materials

Supplementary Figure 1: Forest plot for univariate Cox regression analysis.

Supplementary Figure 2: “Leave-one-out” sensitivity analysis for the genetic causal associations with IBS against PD.

Supplementary Figure 3: Scatter plot of the genetic causal associations with IBS against PD using different MR methods.

Supplementary Figure 4: Funnel plot of the genetic causal associations with IBS against PD using method of inverse variance weighted and MR Egger.

Supplementary Figure 5: Forest plot for the genetic causal associations with IBS against PD.

Supplementary Figure 6: Instrumental variable selection method for Mendelian randomization.

Supplementary Table 1: ICD-10 codes used for identification of exclusive diagnoses.

Supplementary Table 2: The Data-Fields and number of participant of variables employed in this study.

Supplementary Table 3:Selected instrumental variables between IBS and PD.

Supplementary Table 4: Baseline characteristics and incidence of Parkinson’s disease in case-control study.

Supplementary Figure 1: Forest plot for univariate Cox regression analysis.

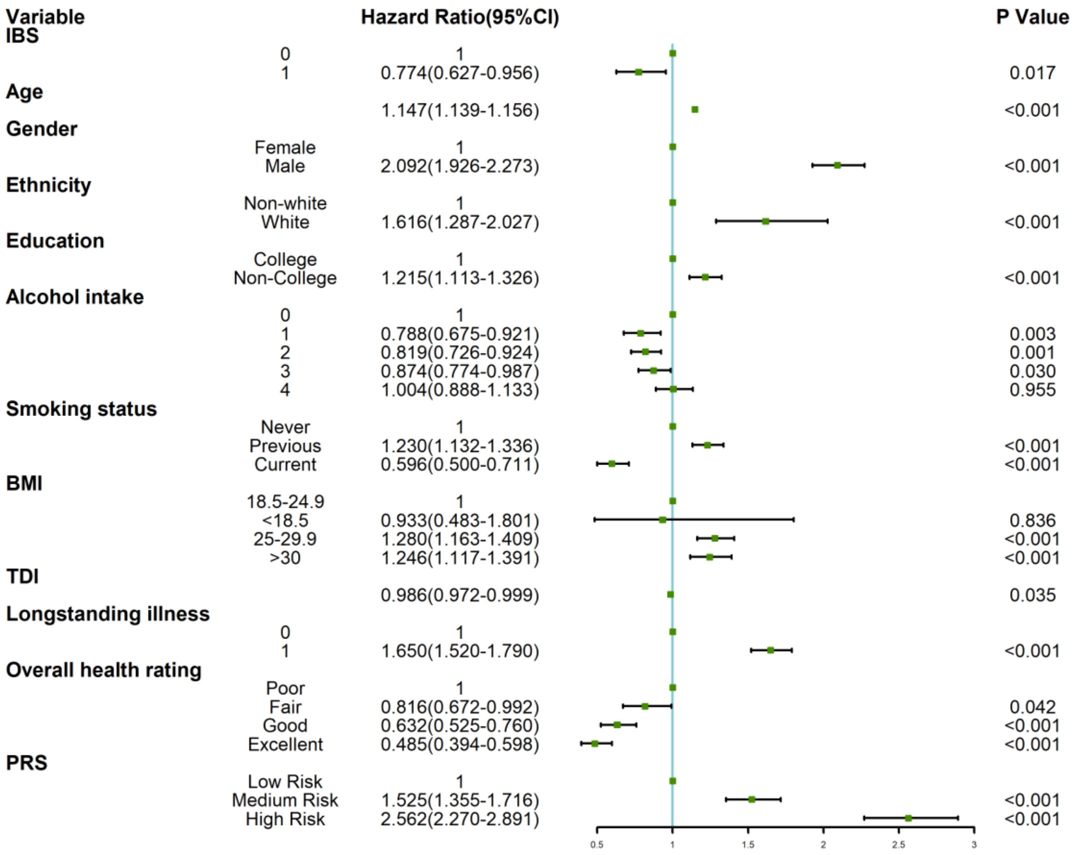

Supplementary Figure 2: “Leave-one-out” sensitivity analysis for the genetic causal associations with IBS against PD.

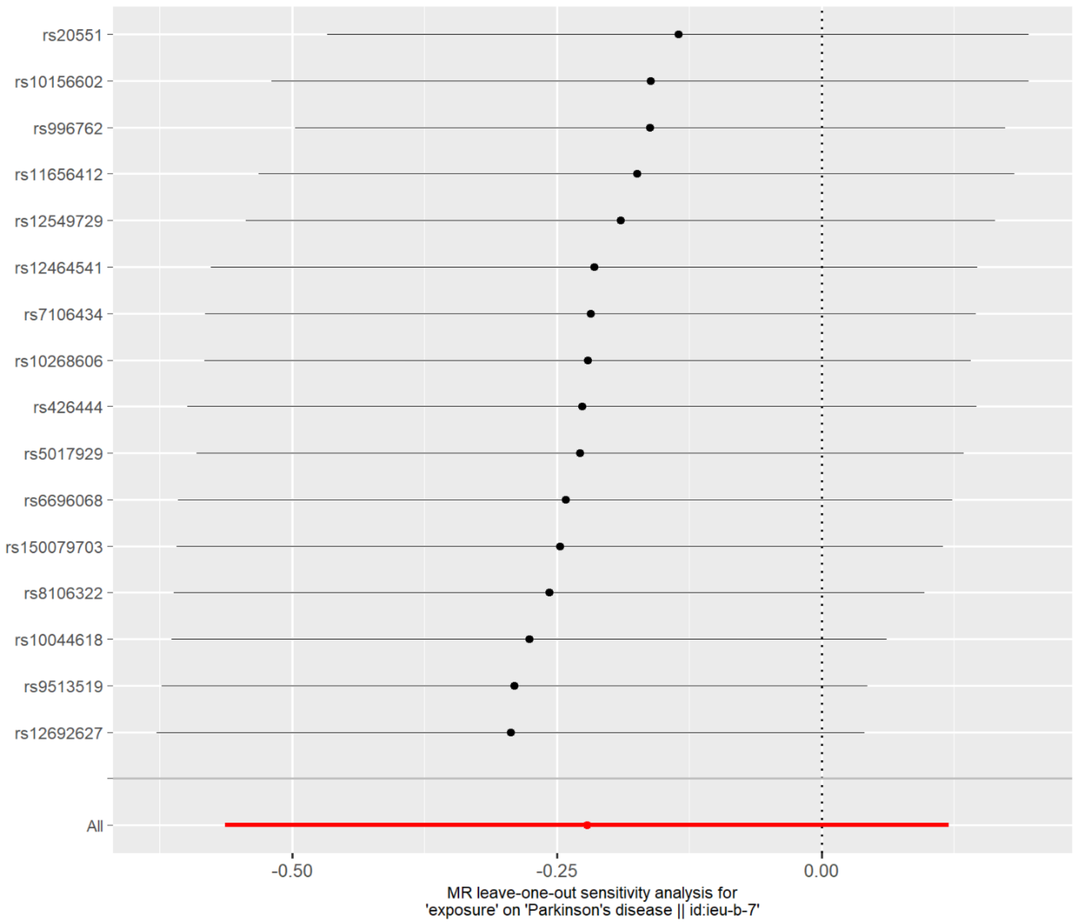

Supplementary Figure 3: Scatter plot of the genetic causal associations with IBS against PD using different MR methods.

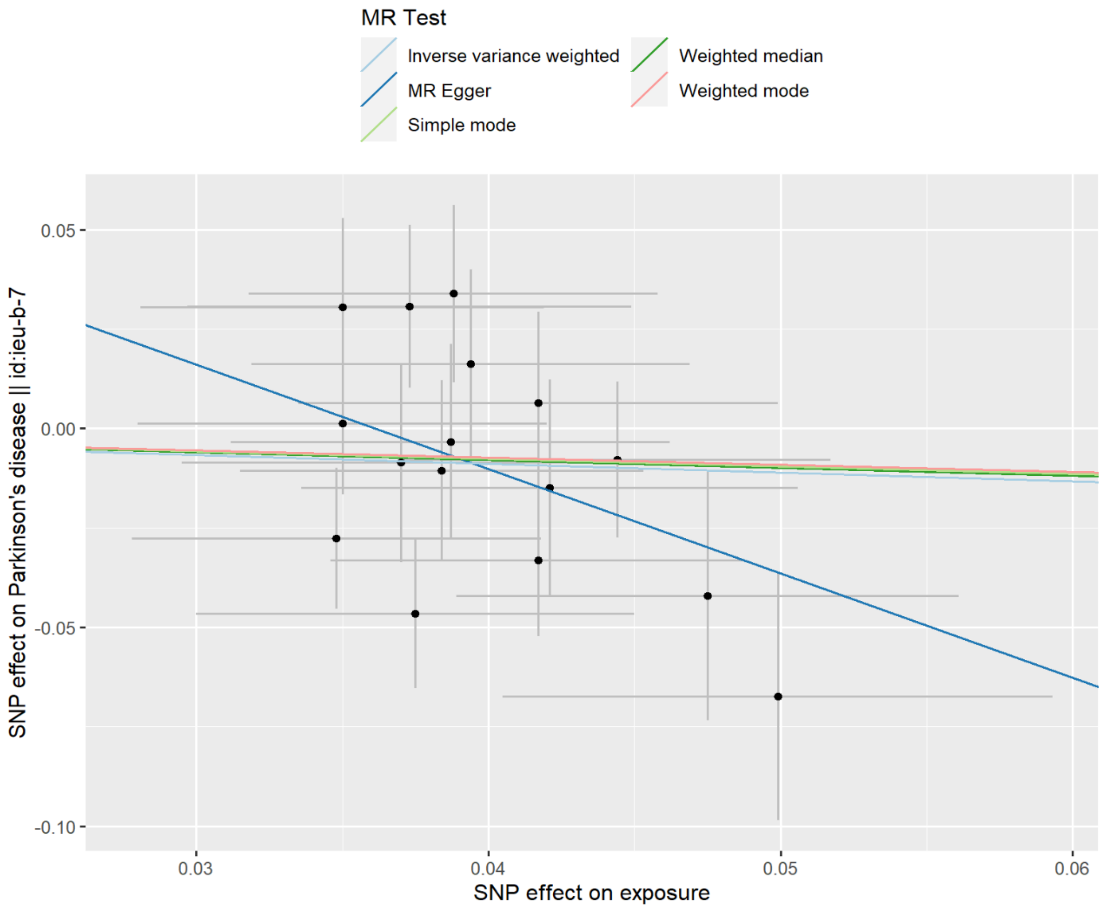

Supplementary Figure 4: Funnel plot of the genetic causal associations with IBS against PD using method of inverse variance weighted and MR Egger.

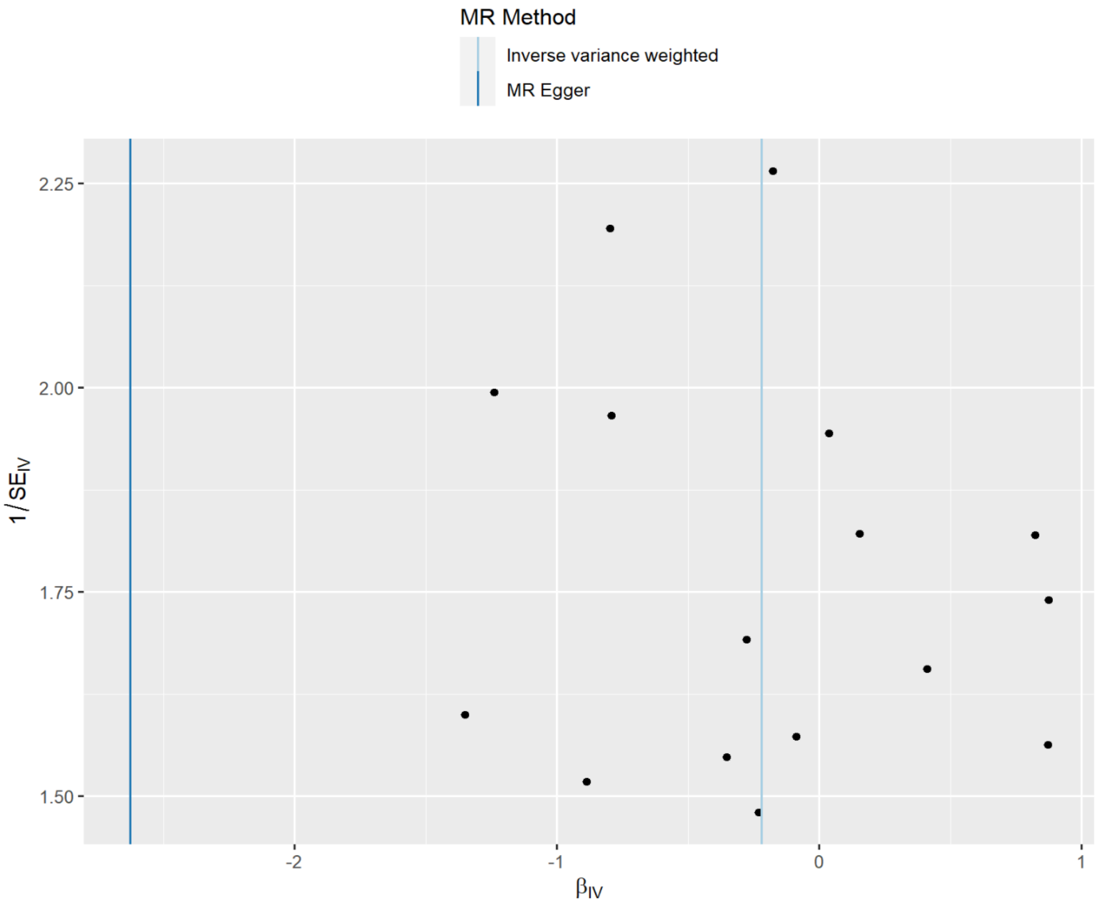

Supplementary Figure 5: Forest plot for the genetic causal associations with IBS against PD.

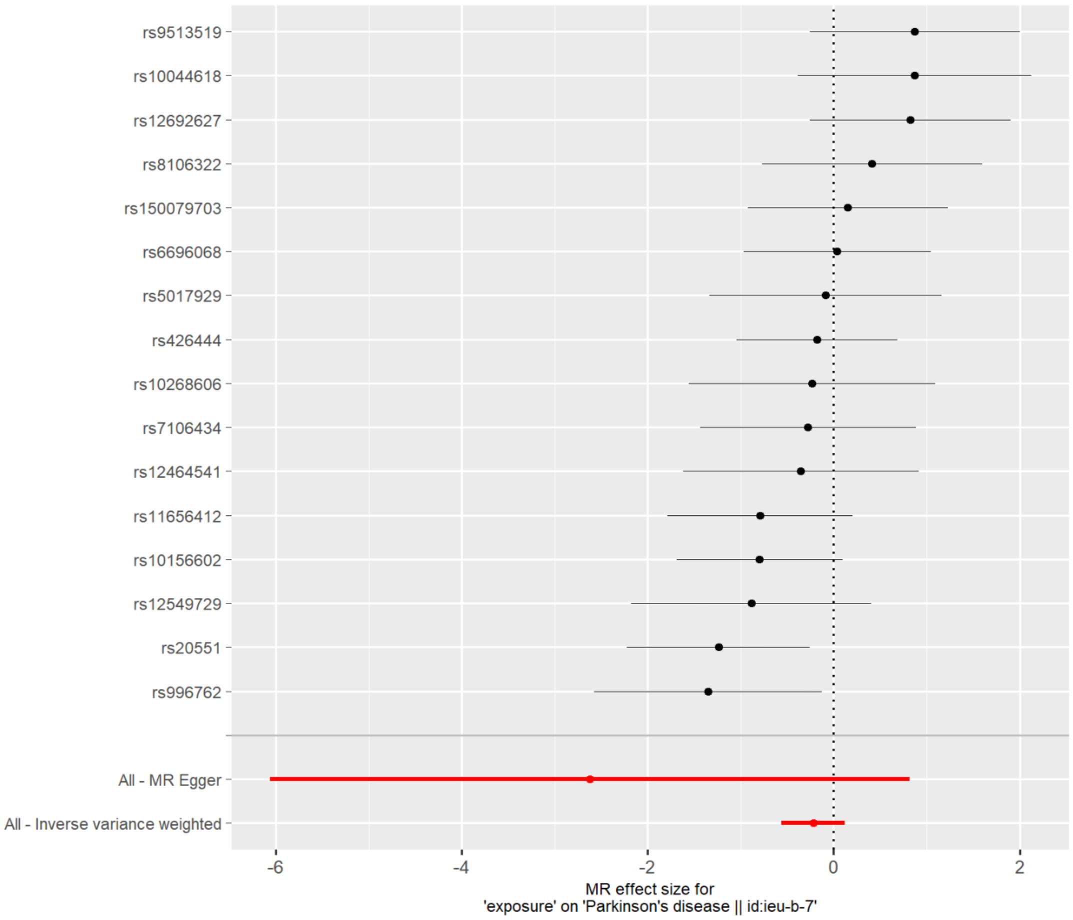

Supplementary Figure 6: Instrumental variable selection method for Mendelian randomization.

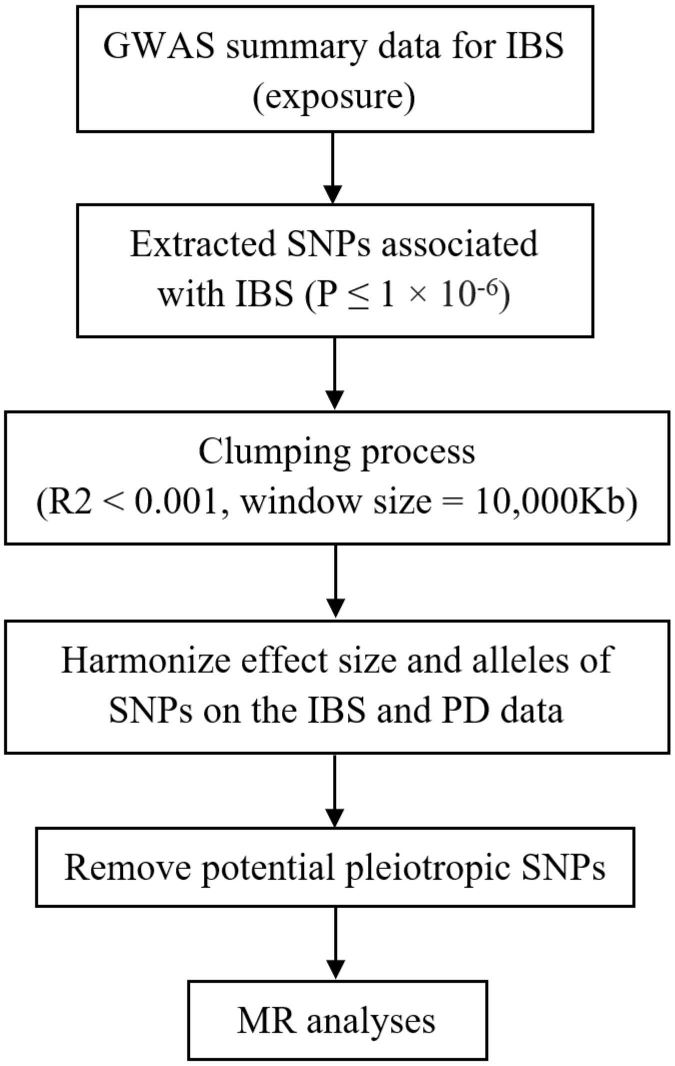

Supplementary Table 1. Selected instrumental variables between IBS and PD.

SNP	Chr	POS	Effect Allele	Other Allele	Exposure (IBS)				Outcome (PD)				
					Beta	eaf	SE	P Value	Beta	eaf	SE	Sample Size	P Value
rs10044618	5	87781168	T	C	0.035	0.4316	0.0069	3.93E-07	0.0305	0.4461	0.0224	468692	0.1733
rs10156602	9	96345328	A	G	0.0417	0.633	0.0071	4.27E-09	-0.0332	0.6378	0.019	482730	0.08085
rs10268606	7	146692467	T	C	0.037	0.704	0.0075	8.08E-07	-0.0086	0.724	0.025	468692	0.730601
rs11656412	17	5155729	A	G	0.0348	0.6096	0.007	6.65E-07	-0.0276	0.6129	0.0177	482730	0.1185
rs12464541	2	82353052	T	C	-0.0421	0.8001	0.0085	7.31E-07	0.0149	0.7882	0.0272	468692	0.585
rs12549729	8	8523912	T	G	0.0475	0.3898	0.0086	3.33E-08	-0.0421	0.3937	0.0313	15976	0.1779
rs12692627	2	161873799	A	G	-0.0373	0.2838	0.0076	9.21E-07	-0.0307	0.2917	0.0205	482730	0.1345
rs13321176	3	150944808	T	G	0.0478	0.8298	0.0092	2.04E-07	-0.1119	0.8267	0.0249	482730	7.23E-06
rs150079703	5	7216226	C	G	0.0417	0.4003	0.0082	3.67E-07	0.0064	0.3819	0.0229	468692	0.780301
rs20551	22	41548008	A	G	-0.0375	0.7149	0.0075	5.73E-07	0.0465	0.7252	0.0188	482730	0.0131801
rs426444	3	84992877	A	T	-0.0444	0.6745	0.0073	1.19E-09	0.0079	0.6785	0.0196	482730	0.6885
rs5017929	8	94653283	A	G	0.0387	0.7009	0.0075	2.47E-07	-0.0034	0.7081	0.0246	468692	0.8894
rs6696068	1	53740797	T	G	-0.035	0.3891	0.007	5.73E-07	-0.0013	0.3695	0.018	482730	0.9424
rs7106434	11	112860579	T	C	0.0384	0.4095	0.0069	2.62E-08	-0.0106	0.418	0.0227	468692	0.6402
rs8106322	19	32024230	A	G	-0.0394	0.646	0.0075	1.49E-07	-0.0162	0.642	0.0238	468692	0.4955
rs9513519	13	99610146	A	G	0.0388	0.6168	0.007	2.98E-08	0.0339	0.6075	0.0223	471013	0.1289
rs996762	2	59772059	C	G	0.0499	0.1576	0.0094	1.11E-07	-0.0673	0.151	0.0312	468692	0.0313502

Supplementary Table 2: Baseline characteristics and incidence of Parkinson's disease in case-control study.

Variable	levels	0 (N=36130)	2 (N=7226)	P Value
Age	Mean ± SD	70.90 ± 8.03	70.90 ± 8.03	1.000
Gender	Female	25675 (71.1%)	5135 (71.1%)	1.000
	Male	10455 (28.9%)	2091 (28.9%)	
Ethnicity	Non-white	1920 (5.3%)	333 (4.6%)	.015
	White	34210 (94.7%)	6893 (95.4%)	
Education	College	12206 (33.8%)	2068 (28.6%)	**<.001**
	Non-university	23924 (66.2%)	5158 (71.4%)	
Alcohol Intake	0	7037 (19.5%)	1705 (23.6%)	**<.001**
	1	4357 (12.1%)	887 (12.3%)	
	2	9655 (26.7%)	1936 (26.8%)	
	3	8193 (22.7%)	1472 (20.4%)	
	4	6888 (19.1%)	1226 (17%)	
Smoking Status	Never	21059 (58.3%)	3947 (54.6%)	**<.001**
	Previous	11789 (32.6%)	2515 (34.8%)	
	Current	3282 (9.1%)	764 (10.6%)	
BMI	18.5-24.9	12585 (34.8%)	2392 (33.1%)	**<.001**
	<18.5	210 (0.6%)	46 (0.6%)	
	25-29.9	14765 (40.9%)	2922 (40.4%)	
	>30	8570 (23.7%)	1866 (25.8%)	
TDI	Mean ± SD	-1.42 ± 3.01	-1.24 ± 3.06	**<.001**
Long standing illness	0	26592 (73.6%)	4361 (60.4%)	**<.001**
	1	9538 (26.4%)	2865 (39.6%)	
Overall health rating	Excellent	6844 (18.9%)	689 (9.5%)	**<.001**
	Fair	6515 (18%)	1977 (27.4%)	
	Good	21796 (60.3%)	3983 (55.1%)	
	Poor	975 (2.7%)	577 (8%)	
PRS	Low Risk	9093 (25.2%)	1794 (24.8%)	.223
	Medium Risk	18160 (50.3%)	3587 (49.6%)	
	High Risk	8877 (24.6%)	1845 (25.5%)	
PD	0	35932 (99.5%)	7177 (99.3%)	.209
	1	198 (0.5%)	49 (0.7%)	

Supplementary Table 3. ICD-10 codes used for identification of exclusive diagnoses.

Exclusive diagnoses	
Crohn' disease	K50
Ulcerative colitis	K51
Other noninfective gastroenteritis and colitis	K52
Celiac disease	K90.0
Colorectal neoplasms	C18, C19, C20, C21, C78.5, D01
Schizophrenia	F20
Secondary parkinsonism	G21
Parkinsonism in diseases classified elsewhere	G22
Other degenerative diseases of basal ganglia	G23
Dystonia	G24
Other extrapyramidal and movement disorders	G25
Multiple system atrophy	G90.3
Progressive vascular leukoencephalopathy	I67.3

Supplementary Table 4. The Data-Fields and number of participant of variables employed in this study.

	Description	Data-Field	Participants	Value Type
1	Age at recruitment	21022	502,357	Inter, years
2	Gender	31	502,357	Categorical(single)
3	Ethnic background	21000	501,472	Categorical(single)
4	Qualifications	6138	498,860	Categorical(single)
5	Alcohol intake frequency	1558	501,487	Categorical (single)
6	Smoking status	20116	501,493	Categorical (single)
7	Body mass index (BMI)	21001	499,401	Continuous, Kg/m2
8	Townsend deprivation index at recruitment	22189	501,731	Continuous
9	Long-standing illness, disability or infirmity	2188	501,466	Categorical (single)
10	Overall health rating	2178	501,466	Categorical (single)
11	Standard PRS for parkinson's disease	26260	486,119	Continuous, relative risk
12	Date G20 first reported (parkinson's disease)	131022	4,571	Date
13	Date K58 first reported (irritable bowel syndrome)	131638	36,219	Date
14	Source of report of K58 (irritable bowel syndrome)	131639	36,219	Categorical (single)
